# First record of the genus *Bankisus* Navás, 1912 in China, with the description of a new species (Neuroptera, Myrmeleontidae)

**DOI:** 10.3897/zookeys.204.3108

**Published:** 2012-06-25

**Authors:** Qingbin Zhan, Xinli Wang

**Affiliations:** 1 Department of Entomology, China Agricultural University, Beijing 100193, China

**Keywords:** Antlion, Myrmeleontiformia

## Abstract

A new species of *Bankisus* Navás, 1912,(*Bankisus sparsus* **sp. n.**)is described and illustrated with the genus newly recorded from China. A key to species of *Bankisus* is provided.

## Introduction

The genus *Bankisus* Navás, 1912 is a small genus of antlions included in the tribe Dendroleontini and occurs in Africa, Yemen and Oman (Stange 2004; Mansell 1985). It is characterized by lack of tibial spurs, long slender legs and hyaline wings with brown markings. Mansell (1985) reviewed the genus and recognized five species: *Bankisus oculatus* Navás, 1912, *Bankisus triguttatus* Navás, 1926, *Bankisus carinifrons* (Esben-Petersen, 1936), *Bankisus elegantulus* (Esben-Petersen, 1936), *Bankisus maculosus* Hölzel, 1983. Recently, Ábrahám (2009) described a sixth species *Bankisus antiatlasensis* from Morocco.Esben-Petersen (1936) placed two species (i.e. *Bankisus carinifrons* and *Bankisus elegantulus*)in his new genus *Navasius* which was subsequently synonymized with *Bankisus* by Markl (1954).

We herein describe the new species, *Bankisus sparsus* sp. n.**,** from China. It is the first *Bankisus* species recorded from outside of Africa and its adjacent area (Yemen, Oman).

## Material and methods

To examine the terminalia, the apex of the abdomen is treated with 10% KOH solution for five-six hours and then transferred to glycerine for further examination. Photographs of partial morphological characteristics were taken by a Canon® EOS 500D digital camera connected with Olympus® U-CTR30-2 microscope and UV-C (Application Suite) applied software by United Vision Ltd. Photographs of habitus were taken by a Nikon® COOLPIX4500 digital camera. All figures were processed in Adobe Photoshop® CS5. Terminology of wing venation follows Wang *et al*. (2003), while female terminalia terminology follows Stange (1994).

## Taxonomy

### Bankisus

Navás, 1912

http://species-id.net/wiki/Bankisus

Bankisus Navás, 1912: 45. Type species: *Bankisus oculatus*, by original designation.
Navasius Esben-Petersen, 1936: 202. Synonymized by Markl 1954: 221.

#### Diagnosis.

Pronotum longer than broad. Wings hyaline with brown marks; Rs arises before CuA fork and three presectoral crossveins before origin of Rs in forewing. Lack of tibial spurs, long slender legs. Female anterior gonapophysis oval or absent.

#### Distribution.

Africa, Oman, Yemen, China.

#### Key to species of *Bankisus*

**Table:** 

1	Forewing 2A and 3A run parallel to each along length	2
–	Forewing 2A and 3A gradually approximate distally along length	4
2	Anterior Banksian line distinct in both wings	3
–	Anterior Banksian line barely discernable in forewing, hindwing without anterior Banksian line	*Bankisus maculosus*
3	Female anterior gonapophysis absent, forewing with few large spots	*Bankisus carinifrons*
–	Female anterior gonapophysis short with dense long brown setae, forewing with many small spots	*Bankisus sparsus* sp. n.
4	Thoracic tergites yellowish	5
–	Thoracic tergites reddish brown	6
5	The spot proximal to hypostigmatic cell extending to costal area hindwing	*Bankisus oculatus*
–	The spot proximal to hypostigmatic cell small, not extending to costal area in hindwing	*Bankisus antiatlasensis*
6	Vertex reddish brown with a row of spots, forewing not much broader than hindwing	*Bankisus elegantulus*
–	Vertex without a row of spots, hindwiwng much narrower than forewing	*Bankisus triguttatus*

### Bankisus sparsus

sp. n.

urn:lsid:zoobank.org:act:804E5172-AE3C-4B80-94E5-D7C1CF79852C

http://species-id.net/wiki/Bankisus_sparsus

Figure 1

#### Diagnosis.

Pronotum yellowish, longer than broad. Wings hyaline with many brown marks, forewing with three presectoral crossveins before origin of Rs, about eight to ten branches of Rs; several discontinuous spots in subcostal area. In hindwing Rs arises before CuA fork, one presectoral crossvein before origin of Rs. Long slender legs, tibial spurs absent. Female terminalia: posterior gonapophysis developed with dense long brown setae; anterior gonapophysis short with dense long brown setae. Lateral gonapophysis developed with dense digging setae.

#### Material examined.

**Holotype**, ♀, China, Guangxi Province, Xiangzhou (23°58.6'N, 109°42.3'E). IX-1980, Fashen Li leg. (CAU-N100208); **Paratype**, sex unknown (abdomen missing), Guangxi Province, Ningming, 180m, (22°07.2'N; 107°04.6'E). 24-V-1984, Fashen Li leg. (CAU-N100209)

The type specimens examined are deposited in the Insect Collections of China Agricultural University (ICCAU), Beijing, China.

***Head*** (Fig. 2)**:** clypeus yellowish; labrum, maxillary and labial palpi yellow; compound eye dark with several black spots, antenna clavate, scape black; vertex inflated, dark with brown spots. ***Thorax*:** pronotum yellowish with several long setae, longer than broad; mesothorax and metathorax black with sparse white long setae. ***Wings*** (Fig. 3)**:** hyaline with many brown marks**,** with several disconnected spots in subcostal area in forewing, Rs arises before CuA fork, three presectoral crossveins and one spot before origin of Rs, about eight branches to Rs, sixteen cross-veins and five spots from origin of Rs to hypostigmatic cell; large brown spot in stigma area; anterior Banksian line discernible and without posterior Banksian lines; ten cross-veins in prefork area. 2A and 3A in the forewings run parallel to each other; a brown oblique stripe in rhegma area and anastomosis of CuA and CuP+1A; hindwing costal area is narrow, Rs arises before CuA fork, one presectoral crossvein before origin of Rs, about eight branches to Rs; anterior Banksian line discernible and without posterior Banksian line; a round spot proximal to hypostigmatic area. ***Legs*** (Fig. 4)**:** long and slender, yellow with black spots and dense setae; claws bent back, sometimes straight. Foreleg: femur yellow brown with white and black bristles, distal part dark; tibia yellowish with dense setae. Tarsus yellow brown except the distal part dark; the first tarsomere long, the fifth slightly shorter; claws bent back; midleg with slightly expanded tibia and claws sometimes straight; hindleg similar to foreleg. ***Abdomen*:** shorter than hindwing, with yellow and black alternate banding. ***Female terminalia*** (Fig. 5-6)**:** ectoproct narrow with dense long setae;anterior gonapophysis short with dense long brown setae; posterior gonapophysis developed with dense long brown setae; lateral gonapophysis with dense digging setae.

#### Distribution.

China (Guangxi Province).

#### Remarks.

*Bankisus sparsus* sp.n. is similar to *Bankisus maculosus* in appearance; however, it can be separated by following characters: forewing with several disconnected spots in subcostal area (*Bankisus maculosus*: without several disconnected spots in subcostal area.); hindwing without spots except two spots at both side of hypostigmatic cell (*Bankisus maculosus*: hindwing with several big spots at apical area).

#### Etymology.

The species name is derived from the Latin *sparsus*, sprinkle; referring to the small spots in forewings.

**Figures 1–6.**
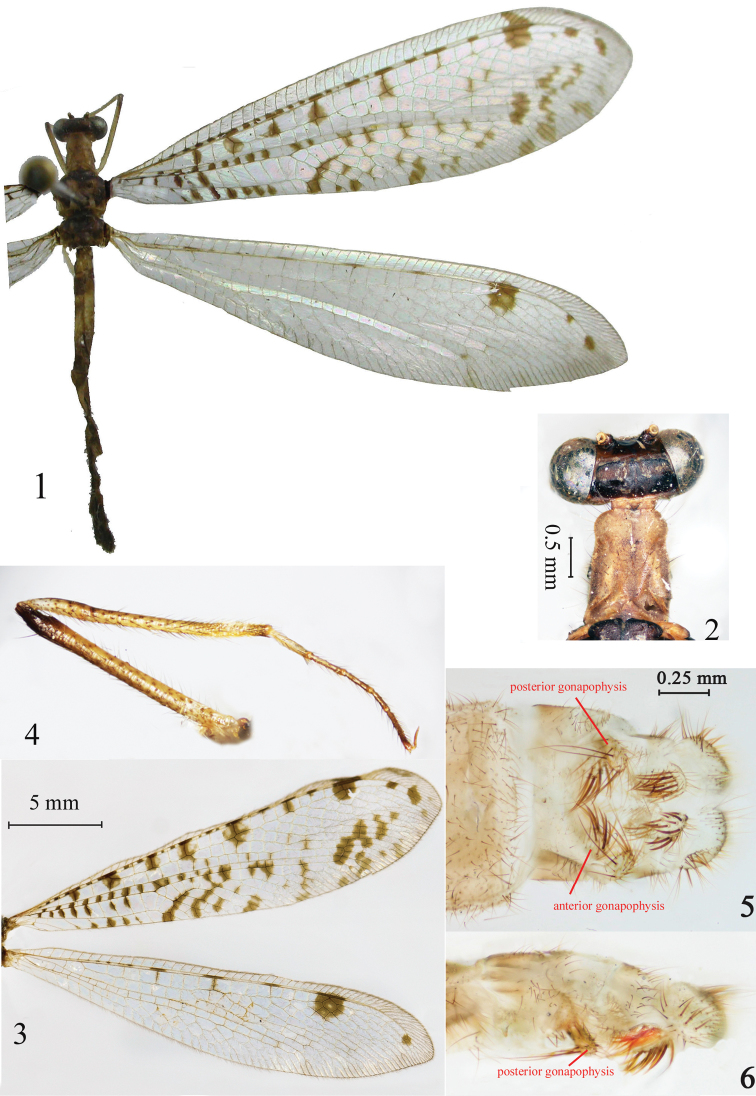
*Bankisus sparsus* sp.n. **1**. Habitus **2** Head and pronotum, dorsal view **3** Wings (Paratype) **4** Foreleg **5–6** Female *terminalia*, ventral and lateral view.

## Supplementary Material

XML Treatment for Bankisus

XML Treatment for Bankisus sparsus
